# CommunityRx, a social care assistance intervention for family and friend caregivers delivered at the point of care: two concurrent blinded randomized controlled trials

**DOI:** 10.1186/s13063-023-07697-z

**Published:** 2023-10-21

**Authors:** Emily M. Abramsohn, MariaDelSol De Ornelas, Soo Borson, Cristianne R. M. Frazier, Charles M. Fuller, Mellissa Grana, Elbert S. Huang, Jyotsna S. Jagai, Jennifer A. Makelarski, Doriane Miller, Dena Schulman-Green, Eva Shiu, Katherine Thompson, Victoria Winslow, Kristen Wroblewski, Stacy Tessler Lindau

**Affiliations:** 1 University of Chicago, 5841 S. Maryland Ave., MC 2050, Chicago, IL 60637 USA; 2 University of Southern California, 1975 Zonal Ave, Los Angeles, CA 90033 USA; 3 New York University, 433 1St Ave, New York, NY 10010 USA

**Keywords:** Randomized controlled trial, Trial design, Innovation, Health-related socioeconomic risk factors, Informational intervention, Community resources, Caregivers

## Abstract

**Background:**

CommunityRx is an evidence-based social care intervention delivered to family and friend caregivers (“caregivers”) at the point of healthcare to address health-related social risks (HRSRs). Two CommunityRx randomized controlled trials (RCTs) are being fielded concurrently on Chicago’s South Side, a predominantly African American/Black community. CommunityRx-Hunger is a double-blind RCT enrolling caregivers of hospitalized children. CommunityRx-Dementia is a single-blind RCT enrolling caregivers of community-residing people with dementia. RCTs with caregivers face recruitment barriers, including caregiver burden and lack of systematic strategies to identify caregivers in clinical settings. COVID-19 pandemic-related visitor restrictions exacerbated these barriers and prompted the need for iteration of the protocols from in-person to remote operations. This study describes these protocols and methods used for successful iteration to overcome barriers.

**Methods and findings:**

CommunityRx uses individual-level data to generate personalized, local community resource referrals for basic, health and caregiving needs. In early 2020, two in-person RCT protocols were pre-tested. In March 2020, when pandemic conditions prohibited face-to-face clinical enrollment, both protocols were iterated to efficient, caregiver-centered remote operations. Iterations were enabled in part by the Automated Randomized Controlled Trial Information-Communication System (ARCTICS), a trial management system innovation engineered to integrate the data collection database (REDCap) with community resource referral (NowPow) and SMS texting (Mosio) platforms. Enabled by engaged Community Advisory Boards and ARCTICS, both RCTs quickly adapted to remote operations. To accommodate these adaptations, launch was delayed until November (CommunityRx-Hunger) and December (CommunityRx-Dementia) 2020. Despite the delay, 65% of all planned participants (CommunityRx-Hunger *n* = 417/640; CommunityRx-Dementia *n* = 222/344) were enrolled by December 2021, halfway through our projected enrollment timeline. Both trials enrolled 13% more participants in the first 12 months than originally projected for in-person enrollment.

**Discussion:**

Our asset-based, community-engaged approach combined with widely accessible institutional and commercial information technologies facilitated rapid migration of in-person trials to remote operations. Remote or hybrid RCT designs for social care interventions may be a viable, scalable alternative to in-person recruitment and intervention delivery protocols, particularly for caregivers and other groups that are under-represented in traditional health services research.

**Trial registration:**

ClinicalTrials.gov: CommunityRx-Hunger (NCT04171999, 11/21/2019); CommunityRx for Caregivers (NCT04146545, 10/31/2019).

**Supplementary Information:**

The online version contains supplementary material available at 10.1186/s13063-023-07697-z.

## Introduction

Family and friend caregivers (“caregivers”) of people with severe or chronic illness are vulnerable to health-related socioeconomic risk factors (HRSRs) like food and housing insecurity and transportation difficulties [1–3]. Clinical trials with caregivers face particular recruitment and retention challenges including caregiver burden [4–6], lack of caregiver identification in medical records [5, 7], and inconsistent caregiver presence during care recipients’ clinical visits [8]. The COVID-19 pandemic exacerbated HRSRs among caregivers [9] and, due to restrictions on caregiver attendance at visits, imperiled caregiver clinical trial enrollment [10].

CommunityRx is an evidence-based social care intervention, informed by self- and family management theory [11], that systematically matches people at the point of healthcare to nearby resources for basic or health-related social needs, wellness, disease self-management, and caregiving needs [12–14]. CommunityRx has been developed and iterated over more than a decade using an asset-based community-engaged approach [13, 15]. Using this approach—which involves solving population health problems by leveraging existing community assets and expertise—CommunityRx was designed for applicability in a wide range of contexts and for a broad spectrum of health and social conditions [12, 13, 15].

CommunityRx-Hunger [16] (*N*_intervention_ = 320, *N*_controls =_ 320) and CommunityRx-Dementia [17] (*N*_intervention_ = 172, *N*_controls_ = 172) are caregiver-centered adaptations of CommunityRx being tested in concurrent randomized controlled trials (RCTs). In both studies, we hypothesize that the intervention will improve caregiver self-efficacy and address HRSRs while not promoting experiences of discrimination nor reducing satisfaction with care. These RCTs are unique in several ways. Both focus on African American/Black caregivers, a population under-represented in research, especially in dementia intervention studies. In addition, both trials are among very few in the social care field to assess outcomes over 12 months [18, 19] (most trials have 3- or 6-month follow-up). Last, few social care trials are blinded. To our knowledge, CommunityRx-Hunger is the first double-blind RCT of a social care intervention. CommunityRx-Dementia is a single-blind trial and is also unique in that it is the first social care intervention study—and one of exceedingly few dementia caregiver studies—to attempt to enroll caregivers at their own point of healthcare.

Before the pandemic, these CommunityRx trials were designed and pre-tested for in-person enrollment and intervention delivery during a child’s hospitalization (CommunityRx-Hunger) or an outpatient visit for a dementia caregiver or their care recipient (CommunityRx-Dementia). To overcome pandemic-related barriers, we rapidly pivoted to remote operations. The purpose of this study is to describe these two unique protocols, in accord with SPIRIT guidelines [20, 21], and replicable strategies we implemented to sustain this research in a predominantly African American/Black community during the COVID-19 pandemic.

## Methods and results

### The CommunityRx Intervention

**Fig. 1:**
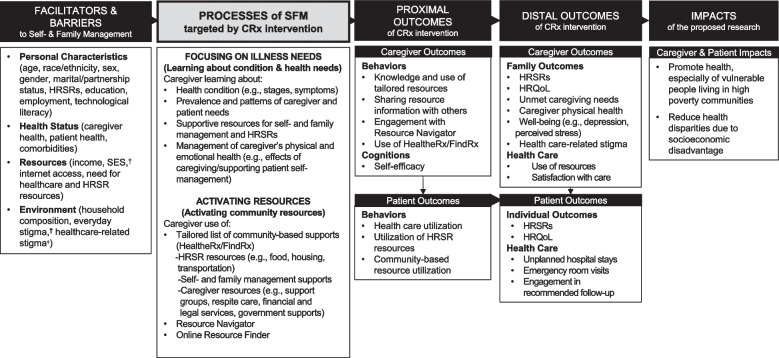
CommunityRx Conceptual Framework, Adapted from the Grey et al. (2015) Self- and Family Management Framework (Grey M, Schulman-Green D, Knafl K, Reynolds NR. A revised Self- and Family Management Framework. Nurs Outlook. 2015 Mar-Apr;63(2):162–70). C, caregiver; P, patient; HRSR, health-related socioeconomic risk factor; HRQoL, health-related quality of life; SFM, self- and family management; SES, socioeconomic status. Dagger symbol (^†^) indicates the following: factors identified by Fundamental Cause Theory as facilitators of or barriers to self- and family management

The conceptual model underlying the CommunityRx interventions was drawn from Grey and colleagues’ Self- and Family Management Framework, an evidence-based framework widely used to develop and test interventions that promote patient and caregiver management of chronic conditions [11, 22]. The framework, which has been applied and iterated for CommunityRx with community input over many years [12], identifies processes of care management associated with patient and family outcomes [23]. These processes underlie specific tasks that comprise the essential work of self- and family management, including learning about health needs and activating resources to address health and other needs [24]. An increasing number of studies, including several of our own community-engaged trials, have applied the framework among racially and ethnically diverse participants [12, 13, 22]. The Framework has been adapted for the CommunityRx-Hunger and -Dementia trials (Fig. 1) to include factors identified by Fundamental Cause Theory (e.g., socioeconomic status and stigma) among other known facilitators and barriers of self- and family management [25–27]. The CommunityRx interventions assist patients and caregivers in learning the skills and resources needed to engage in these processes with confidence (self-efficacy) [28].

**Fig. 2:**
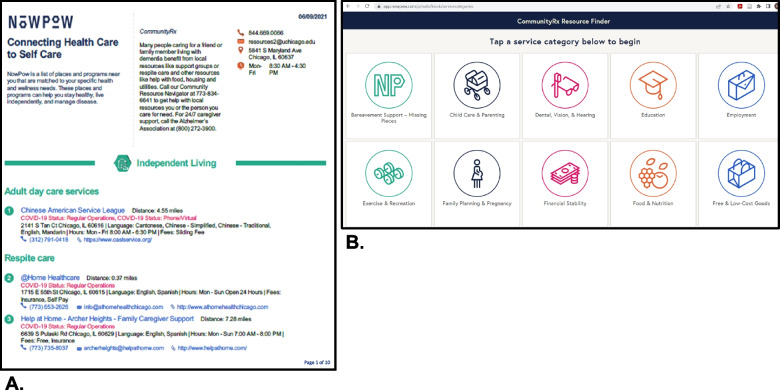
Example from CommunityRx-Dementia of the HealtheRx resource list (**A**) and FindRx tool (**B**). **A** A sample “HealtheRx” resource list for CommunityRx-Dementia, generated by NowPow and facilitated by ARCTICS. **B** Home page of Community Resource Finder Tool, “FindRx,” for CommunityRx-Dementia

**Fig. 3:**
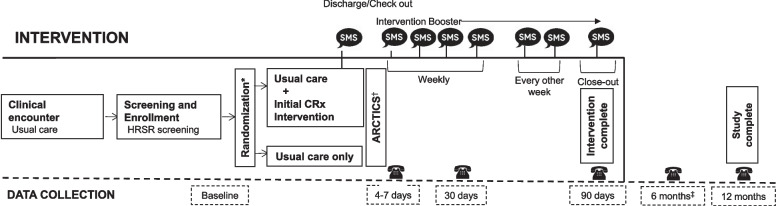
CommunityRx RCT enrollment, intervention, and data collection timeline. Asterisk symbol (*) indicates the following: stratified randomization by health-related social risk factors (HRSRs) was employed in both studies: CommunityRx-Hunger was stratified on food security status (food secure versus food insecure) and CommunityRx-Dementia was stratified by number of HRSRs (0 HRSRs versus ≥ 1 HRSR). Dagger symbol (^†^) indicates the following: RCT data flows to and through the Automated Randomized Controlled Trial Intervention-Communication System (ARCTICS) that facilitates generation and delivery of the CommunityRx intervention and survey reminders (see Fig. 4). Double dagger symbol (^‡^) indicates the following: 6-month follow-up survey specific to CommunityRx-Hunger

Accordingly, the CommunityRx-Hunger and -Dementia interventions are comprised of three, evidence-based components that target key self- and family management processes: (a) education about the prevalence of HRSRs among caregivers; (b) activation of resources through delivery of and coaching on how to use a personalized resource “prescription” (HealtheRx, Fig. 2A); and (c) boosting of the intervention through a series of proactive text messages and ongoing navigator support. The CommunityRx intervention is delivered by a navigator (a research assistant) at the index clinical encounter—either prior to a child’s hospital discharge (CommunityRx-Hunger) or following an outpatient visit (CommunityRx-Dementia). Initial intervention delivery includes the education and activation components of the intervention. Boosters are delivered over three months and caregiver outcomes are assessed over 12 months (Fig. 3).

#### Education

In both trials, the navigator, using a brief semi-structured script, provides caregivers with information about the prevalence of HRSRs among caregivers (to reduce stigma) and the availability of nearby community resources for assistance. The scripts for each study were developed with advisory board input and convey messages such as “caregivers can benefit from resources” and “caring for yourself allows you to better care for your loved ones.” The scripts were designed to allow for personalization. An abridged version of the script is included on the HealtheRx resource list (Fig. 2A).

#### Resource activation

The activation component of the interventions aims to promote caregiver resource use through navigator-led coaching. The navigator reviews the HealtheRx with the caregiver and explains how to access resources by pointing out key resources and features (e.g., insurance accepted, hours). Caregivers are instructed to contact the navigator by text, email, or phone to find additional resources. When contacted, the navigator then searches the resource referral platform to identify and share resources using the caregiver’s preferred delivery mode (email or text message).

A new component, “FindRx,” was added to the CommunityRx-Dementia design: the navigator uses a web-enabled tablet to demonstrate this client-facing resource finder (Fig. 2B). The navigator coaches caregivers on how to use FindRx to search for and share community resources, request additional resource information, and give feedback about resources.

#### Boosting

The interventions are “boosted” over 3 months with a series of automated text messages from the navigator offering caregivers ongoing support and resource information. The timing and frequency of these messages draws on evidence from the Critical Time Intervention model, which recognizes transitions in care (e.g., from hospital or clinic to home) as highly influential or teachable moments [29]. The Critical Time Intervention model uses a phased intervention approach with more frequent touchpoints early in the intervention that become less frequent over time (Fig. 3). The content of these text messages was designed to promote sustained engagement with the navigator [30] and provide caregivers with ongoing community resource information and navigational support. A prior observational study found that adding a text message component to the CommunityRx intervention increased participant engagement with the navigator by 70 fold (0.2 to 14%) [13].

In each trial, the CommunityRx intervention is compared to usual care, which includes typical inpatient (CommunityRx-Hunger) and outpatient (CommunityRx-Caregiver) procedures such as meeting with a patient service representative, receipt of a printed after visit summary (AVS), scheduling of future visits and social work referral at the discretion of the healthcare team. In addition to these typical procedures, usual care for CommunityRx-Hunger includes information about all available retail food options in the hospital, including the Feed1st food pantry program. Feed1st, in partnership with a regional food depository, hospital and medical student volunteers and others, operates multiple open-access, self-serve food pantry sites at the same site as the CommunityRx trials. The pantry sites are open 24/7/365, and food is free for anyone in the hospital with no questions asked [31–33]. Usual care in both settings can also include provision of community resource information, but delivery of this information is neither systematic nor comprehensive. No other interventions or procedures associated with usual care were prohibited during the time of the trials.

### Clinical trial design: community engagement and innovation

The design characteristics, scientific aims, and outcomes of both CommunityRx RCTs are outlined in Table 1. Each study uses stratified randomization to enroll potential participants at the point of care and assign caregivers to usual care or the intervention in a 1:1 ratio. Stratified randomization used the method of permuted blocks with blocks of varying sizes and the uniform random number function in Stata version 16 and facilitated using the randomization function in REDCap. Important caregiver and patient outcomes are assessed at multiple time points over 12 months following enrollment (Table 2). Following 12-month data collection, eligible participants in each trial will be invited to participate in a qualitative interview to elicit their perspective on the intervention, among other attitudes and beliefs. These qualitative data will be used to help interpret and triangulate the quantitative survey data. All data, including protected health information (PHI) are collected in REDCap, a password-protected, HIPAA compliant survey database only accessible to approved study researchers, who regularly monitor all protocols. Participants in each study provide documentation of informed consent, including permission to re-contact them for future research opportunities (Supplements 2 and 3). Both trials and all protocol amendments were approved by the study site’s Institutional Review Board (IRB) and deemed “minimal risk.” Consequently, data and safety monitoring is performed by study statisticians (rather than an external monitoring board). Per regulatory guidelines, any adverse events would be reported to the Institutional Review Board (IRB).

**Table 1:** Design characteristics, scientific aims, and corresponding outcomes of two CommunityRx clinical trials

	**CommunityRx-Hunger** (protocol date November 16, 2020)	**CommunityRx-Dementia** (protocol date November 13, 2020)
**Study design**	Double-blind randomized controlled trial (both researcher and participant blinded to study arm)	Single-blind randomized controlled trial (participant blinded to study arm)
**Study population**	Parents and caregivers of hospitalized children (*N* = 640)	Family and friend caregivers of home-dwelling people with Alzheimer’s disease and related dementias (*N* = 344)
**Setting**	Inpatient units at an urban academic children’s hospital	Adult outpatient clinics at an urban academic medical center
**Inclusion criteria**	• Self-identifies as the primary caregiver of a child younger than 18 years of age admitted to the children’s hospital • English- or Spanish-speaking • Resides in a target ZIP code • Access to a cell phone and willing to use for research participation • Agrees to receive text messages from the study	• Self-identifies as a caregiver of a home-dwelling person with Alzheimer’s Disease or related dementia using an adaptation of the BRFSS^a^ caregiver module • Resides in a target ZIP code • Access to a cell phone willing to use for research participation • Agrees to receive text messages from the study
**Exclusion criteria**	• Minor caregivers who are not emancipated minors according to Illinois State law • Non-parental minor caregivers • Caregivers of hospitalized healthy newborns • Caregivers of children who are admitted for less than 24 hours • Caregivers of children hospitalized at index hospitalization with a diagnosis of disordered eating • Enrollment in pre-test	• Recalls participating in CommunityRx in the past • Minor caregivers who are not emancipated minors according to Illinois State law • Enrollment in pre-test
**Screening**	USDA 18-item food security screener^b^ (prior 12 months)	CMS AHC 10-item HRSR screener^c^
**Stratification**	Food secure (score of 0–2) vs. food insecure (score of ≥ 3)^b^	HRSR status (no HRSRs vs. one or more HRSRs)
**Randomization**	1:1 randomization, stratified by food security status	1:1 randomization, stratified by HRSR status
**Usual care**	Information from hospital staff including available food options in the hospital and the Feed1st food pantry program, regular visits from Child Life Services and referral to social work (if appropriate)	Information from hospital staff, which may include transmission of information about community resources
**Survey Timepoints**	Baseline, 1W, 1 M, 3 M, 6 M, 12 M post-discharge	Baseline, 1W, 1 M, 3 M, 12 M post-index clinical encounter
**Study endpoint**	12 M	12 M
**Scientific aims with primary and secondary outcomes**	*Aim 1*: Among caregivers of hospitalized children experiencing household food insecurity, evaluate the longitudinal effects of CommunityRx-Hunger versus usual care on self-efficacy for finding resources (primary outcome), severity of household food insecurity, adult and child nutrition and health, and child healthcare utilization (secondary outcomes)	*Aim 1*: Among dementia caregivers with unmet HRSNs, evaluate the longitudinal effects of CommunityRx-Dementia versus usual care on caregiver self-efficacy (primary outcome) and psychosocial (unmet needs, social isolation, well-being, burden, depression, stress), behavioral (community resource use), health and healthcare utilization (secondary outcomes)
	*Aim 2*: Among all caregivers of hospitalized children, evaluate the effects of CommunityRx-Hunger versus usual care on caregiver satisfaction with care (primary outcome), caregiver and child health and caregiver stigma during hospitalization (secondary outcomes)	*Aim 2*: Among all dementia caregivers, evaluate the effects of CommunityRx-Dementia versus usual care on the healthcare experience, including satisfaction with care (primary outcome), experiences of stigma during clinical care and likelihood of sharing community resource information with others

*Abbreviations*: *AHC* Accountable Health Communities, *BRFSS* Behavioral Risk Factor Surveillance System, *CMS* Centers for Medicare & Medicaid Services, *HRSRs* Health-related social risks, *M* month, *W* week

^a^Centers for Disease Control and Prevention (CDC). Behavioral Risk Factor Surveillance System Caregiver Module. https://www.cdc.gov/aging/healthybrain/brfss-faq-caregiver.html

^b^U.S. Department of Agriculture. U.S. Household Food Security Survey Module (18-item). https://www.ers.usda.gov/topics/food-nutrition-assistance/food-security-in-the-us/survey-tools/

^c^Centers for Medicare and Medicaid Services. The Accountable Health Communities Health-Related Social Needs Screening Tool. https://innovation.cms.gov/files/worksheets/ahcm-screeningtool.pdf

**Table 2:** Key caregiver and patient outcomes and survey timepoints, by study

	**CommunityRx-Hunger**							**CommunityRx-Dementia**						
	**Timepoints**													**Measures and survey instruments**
**Key outcomes**	S	BL	1W	1 M	3 M	6 M	12 M	S	BL	1W	1 M	3 M	12 M	
Food security (12 M)	x						x							USDA 18-item HFSS^a^
Food security (30 day)		x		x	x	x	x							Adapted USDA 18-item HFSS^a^
HRSRs			x				x	x	x		x	x	x	CMS AHC 10-item Social Needs Screening Tool^b^
Self-Efficacy for Finding Resources		x	x	x	x	x	x		x		x	x	x	Adapted Bandura Self-Efficacy Scale^c^
Caregiving Self-Efficacy									x		x	x	x	Caregiver Care and Self-Efficacy Survey, Self-Efficacy for Caregiving subdomain^d^
Satisfaction with Care										x				Patient Satisfaction Questionnaire^**e**^
Satisfaction with Care, Discharge			x											Child HCAHPS^f^
Healthcare-Related Stigma			x							x				Discrimination in Medical Settings Scale^g^

*Abbreviations*: *AHC* Accountable Health Communities, *BL* baseline, *CMS* Centers for Medicare & Medicaid Services, *HCAHPS* Hospital Consumer Assessment of Healthcare Providers Survey, *HFSS* Household Food Security Survey, *HRSR* health-related social risk, *M* month, *S* screener, *USDA* United States Department of Agriculture, *W* week

^a^U.S. Department of Agriculture. U.S. Household Food Security Survey Module (18-item). https://www.ers.usda.gov/topics/food-nutrition-assistance/food-security-in-the-us/survey-tools/

^b^Centers for Medicare and Medicaid Services. The Accountable Health Communities Health-Related Social Needs Screening Tool. https://innovation.cms.gov/files/worksheets/ahcm-screeningtool.pdf

^c^Bandura A. Self efficacy: the exercise of control. New York: WH Freeman; 1997

^d^Jennings LA, Reuben DB, Evertson LC, Serrano KS, Ercoli L, Grill J, Chodosh J, Tan Z, Wenger NS. Unmet needs of caregivers of individuals referred to a dementia care program. J Am Geriatr Soc. 2015 Feb;63(2):282–289. PMCID: PMC4332558

^e^Marshall GN, Hays RD. The Patient Satisfaction Questionnaire Short-Form (PSQ-18). Santa Monica, CA: RAND Corporation, 1994. https://www.rand.org/pubs/papers/P7865.html

^f^Toomey SL, Zaslavsky AM, Elliott MN, Gallagher PM, Fowler FJ, Klein DJ, Shulman S, Ratner J, McGovern C, LeBlanc JL, Schuster MA. The development of a pediatric inpatient experience of care measure: Child HCAHPS. Pediatrics. 2015 Aug;136(2):360–369

^g^Peek ME, Nunez-Smith M, Drum M, Lewis TT. Adapting the everyday discrimination scale to medical settings: Reliability and validity testing in a sample of African American patients. Ethn Dis. 2011;21(4):502–509

#### Enrollment sites

CommunityRx-Hunger enrolls parents/caregivers during a child’s hospitalization at an urban academic children’s hospital. CommunityRx-Dementia enrolls family/friend caregivers at the point of their own outpatient healthcare or during outpatient healthcare visits of their care recipient at a large, urban academic medical center serving a densely populated 110mi^2^ urban area, including one of the largest contiguous African American/Black urban communities in the USA. Here, 49% of people have an annual household income < 200% of the federal poverty level. Seventy-six percent of residents in the hospital’s Primary Service Area are African American/Black, and 13% are Hispanic [34].

#### Community engagement: theory, background, and methods

CommunityRx uses an asset-based, community-engaged approach to research, which involves working with community members and organizations to achieve locally relevant scientific objectives [15, 35]. Both RCTs involve community advisory boards (CABs) composed of community and clinical stakeholders, including caregivers, patients, clinicians, hospital staff, community advocacy organizations, and volunteers. CABs convene regularly to review and advise on study protocols, intervention design, and dissemination efforts. CAB members who are not employees of the academic medical center are compensated.

CommunityRx-Hunger is advised by the Feed1st CAB, a group originally formed to work with researchers to combat high rates of food insecurity among people seeking healthcare, including parents and other caregivers with children admitted to our children’s hospital [31, 32]. During the pandemic, Feed1st launched 5 new pantry sites, including one new pantry in the children’s hospital (CommunityRx-Hunger trial site) and two new pantries in outpatient settings where we are enrolling for the CommunityRx-Dementia trial. As these two trials frequently identify people with food insecurity, Feed1st is essential to the ethical conduct of research in our setting.

The CommunityRx-Dementia CAB grew from a group originally established to advise the Supporting Healthy Aging Resources and Education (SHARE) Network, a Health Resources and Services Administration-funded program with a large network of community-based organizations serving older adults with and without dementia in the CommunityRx study area [36]. During the pandemic, to adjust to remote operations, CAB members recommended steps to ensure trial accessibility for caregivers with low technology literacy, connecting us to Tech Savvy Friends [37], a medical student-led organization that provided technical support to caregivers who needed help with enrollment tasks, such as creating and accessing a personal email address and opening and navigating web links. Both CABs played an essential role in ensuring that changes to remote operations were caregiver-centered.

#### ARCTICS: Automated Randomized Controlled Trial Intervention-Communication System

**Fig. 4:**
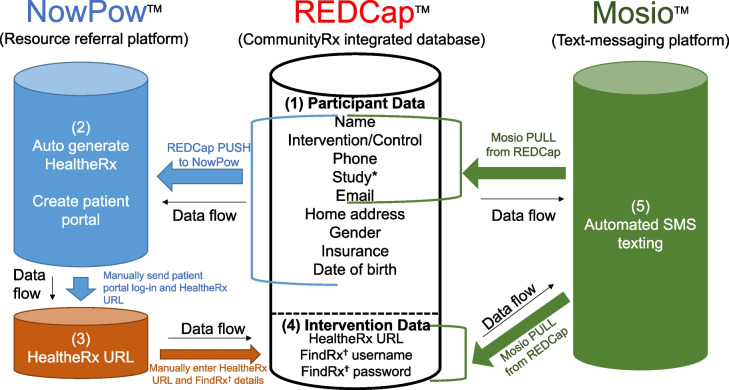
Automated Randomized Controlled Trial Intervention-Communication System (ARCTICS). Asterisk symbol (*) indicates the following: study refers to the clinical trial in which the individual is enrolled (e.g., CommunityRx-Hunger, CommunityRx-Dementia). Dagger symbol (^†^) indicates the following: FindRx is specific to the CommunityRx-Dementia intervention

Funding for both trials was awarded around the same time by different institutes at the National Institutes of Health (CommunityRx-Hunger by the National Institute on Minority Health and Health Disparities and CommunityRx-Dementia by the National Institute on Aging). Although not proposed in either application, we saw an innovation opportunity that would enable us to realize operational efficiency and minimize burden on participants. Drawing on experience developing the CommunityRx information technology platform and integrating it with EMR systems [13], we created the Automated Randomized Controlled Trial Intervention-Communication System (ARCTICS), a novel application programming interface (API) and a custom middleware to enable interoperability of the survey database (REDCap [38, 39]) with the community resource referral (NowPow [40]) and text messaging (Mosio [41]) platforms (Fig. 4) [42]. ARCTICS, developed in collaboration with the academic medical center's research informatics team, draws on individual-level demographic, health and social risk data captured via REDCap-administered surveys to facilitate generation and delivery of (a) personalized resource referrals (HealtheRxs) and (b) text messages to participants for intervention information, survey reminders, retention, and scheduling.

### Iteration of the trial protocols for remote operation

Research protocols for both trials were initially planned for in-person administration and pre-tested in-person in January 2020. Following the declaration of the COVID-19 pandemic on March 11, 2020, clinical trials involving in-person activities outside of routine care at the medical center were suspended [43]. Furthermore, adult outpatients could not be accompanied by a caregiver for visits and hospitalized children were restricted to one parent/caregiver. Accordingly, we revised our protocols to enable remote recruitment, enrollment and intervention. These efforts were enabled by ARCTICS and an engaged clinical staff and were carried out with input from each study’s CAB.

#### Recruitment

Because we could no longer recruit caregivers in person, protocols were iterated to allow for contact via phone and text message. Researchers used demographic and emergency contact information in patients’ electronic medical records (EMR) to identify potential caregivers and facilitate recruitment. Compared to in-person pre-test data, remote pre-test data showed improvements in approach and enrollment rates in both RCTs.

For CommunityRx-Hunger, the pre-test approach rate (the number of caregivers we were able to contact to assess interest in the study over the total number of caregivers identified to approach) increased from 33% (at hospital bedside) to 54% using remote protocols. With input from the Feed1st CAB, the recruitment protocol for CommunityRx-Hunger was further modified for the full RCT to a three-pronged approach: (1) call to phone at hospital bedside; (2) text message to parent’s cell phone listed in their child’s EMR; and (3) follow-up phone call to caregiver’s cell phone.

For CommunityRx-Dementia, researchers attempted to approach all patients awaiting a visit in the target clinics during the in-person pre-test period. More than 1000 individuals (*N* = 1037) were approached in person to assess their dementia caregiver status, ultimately enrolling 10 caregivers for this pre-test. A true approach rate cannot be calculated for this recruitment strategy because we were unable to ascertain the full count of patients awaiting care during this time. To adjust to pandemic conditions, this recruitment protocol was iterated for remote recruitment by leveraging our EMR data warehouse and informatics innovations to help target recruitment of caregivers at their own point of care and those providing care to patients with dementia seen in our healthcare system (the EMR does not identify individuals as caregivers, either in patients with dementia or individuals’ own medical records). Remote recruitment protocols included sending an introductory text message to the cell phone of the patient’s emergency contact person (listed in the EMR) and then proceeding to call and leaving a voicemail as needed. Of 752 patients identified, 365 were approached remotely to assess their dementia caregiver status, ultimately enrolling 10 caregivers for the pretest.

#### Screening

In the original, in-person protocols, screening for food insecurity and other HRSRs was self-administered on a tablet at the point of care. To maintain privacy in the remote protocol, screening was conducted by phone. The percentage of caregivers screened for inclusion among those approached was 51% for CommunityRx-Hunger (versus 56% during in-person recruitment) and 68% for CommunityRx-Dementia (versus 57%).

#### Enrollment

Following remote screening, caregivers were emailed or texted a link to the informed consent document. Researchers conducted the informed consent process by phone while the caregiver reviewed the form on their own device. Informed consent was documented electronically using REDCap’s e-consent Framework in accordance with FDA rule 21 CFR [38, 39, 44]. The percentage of caregivers who consented among those who were eligible was lower using remote compared to in-person protocols: remote 69% (22/32) vs. in-person 77% (10/13) for CommunityRx-Hunger and remote 71% (10/14) vs. in-person 77% (10/13) for CommunityRx-Dementia. Following consent, baseline data were collected by phone or videoconference.

Both studies were projected to launch by March 2020 and complete by December 2021 (22 months). The pandemic caused a ~ 9-month delay: CommunityRx-Hunger launched in November and CommunityRx-Dementia in December 2020. With no additional funding or time allotted for recruitment, both studies enrolled 65% of their pre-pandemic targets in the first 9 months of a shortened (18 month) enrollment timeline and 13% more participants than projected over the first 12 months of enrollment. Additionally, shifting to remote protocols did not jeopardize the diversity of our projected sample of caregivers. In fact, in both trials, we enrolled more African American or Black caregivers using remote protocols than originally estimated using in-person protocols (CommunityRx-Hunger: 58% in-person versus 78% currently in the RCT; CommunityRx-Dementia: 75% in-person versus 86% currently in the RCT).

#### Intervention delivery

Following enrollment and baseline data collection, participants were stratified by HRSR status (food secure versus food insecure for CommunityRx-Hunger and 0 HRSRs versus ≥ 1 HRSR for CommunityRx-Dementia) and randomized to either usual care or the CommunityRx intervention. The pivot to remote operations required major changes to the intervention delivery protocol that were facilitated by ARCTICS and rapid, pandemic-related uptake of videoconferencing by healthcare professionals and lay caregivers alike.

To simulate the brief in-person, face-to-face encounter that was originally planned for intervention delivery, we implemented videoconferencing. Using data that flowed through ARCTICS, navigators quickly generated a personalized HealtheRx for each caregiver. The navigator used the videoconferencing screen-sharing feature to coach the caregiver on how to use the HealtheRx (and, for CommunityRx-Dementia, how to use the online FindRx tool). When videoconferencing was infeasible (for example, the caregiver was not in the child’s hospital room to receive a tablet or did not have videoconferencing capabilities on their own device), we used phone. All caregivers, regardless of how the initial intervention was delivered, received the HealtheRx by email and text message to their mobile phone. For CommunityRx-Dementia participants, FindRx information was sent via email within a week of the index outpatient visit. This email included the caregiver’s unique login information for FindRx, a brief visual user guide with instructions on how to use the FindRx tool, a 6-min video tutorial link and contact information for the navigator. The text message protocol remained the same as described above.

For CommunityRx-Hunger, we engaged specialists from hospital Child Life Services (CLS) to support remote intervention delivery. CLS specialists are trained professionals who routinely interact in person with patients and families before hospital discharge to help them understand their illnesses and procedures through expressive therapies, medical education, and other support, often using tablets and other information technologies [45]. Given limits on family support at the bedside, CLS played a critical role in supporting hospitalized children during the pandemic [46, 47]. Following randomization, CLS specialists were dispatched by the research team to deliver a web-enabled tablet to the caregiver at a scheduled date and time prior to the patient’s discharge. They also provided technical support to the caregiver for videoconferencing. For CommunityRx-Dementia, caregivers who had trouble accessing a personal email or opening web links on their cell phone were referred to Tech Savvy Friends [37] before consenting to the study. After the caregiver received support from Tech Savvy Friends, they were re-contacted by the data collector to complete enrollment.

#### Data collection, retention, and monitoring

Item missingness in the in-person pre-test ranged from 0 to 13% at baseline and 1 week and was 0% for the remote pre-test. Most retention strategies remained the same when moving from in-person to remote protocols, including the use of text messages and scheduled calls to facilitate follow-up survey completion. However, new strategies were implemented to promote retention over 12 months of follow-up. For CommunityRx-Hunger, a text message to participants between their 6- and 12-month surveys reminded study participants of their upcoming survey and confirmed their contact information. CommunityRx-Dementia implemented a 6- and 9-month check-in to facilitate retention and confirm contact information. All participants who verified their contact information were entered into a quarterly raffle in which 2 winners received a $50 gift card. CommunityRx-Dementia also implemented a graduated incentive structure wherein the compensation for each completed survey increased over time. Once enrolled, there were no criteria for discontinuing study participants. However, caregivers were able to withdraw from the study at their discretion.

To maintain blinding, only approved researchers had access to the unblinded data for the purposes of trial monitoring and analysis. To ensure data quality, item missingness, range checks, and item non-response were assessed by study analysts regularly for both trials. Data monitoring, preparation, and dissemination were ongoing throughout the study periods. Trial conduct and progress were regularly reported to the study sponsor and institutional review board. Both trials were registered on ClinicalTrials.gov in accordance with NIH policy and reflect all items from the WHO Trial Registration Data Set. All reporting complies with CONSORT guidelines [48].

#### Data management and statistical analysis

Data management was facilitated and data securely stored using REDCap, managed by the academic medical center's research informatics team.  Stratified randomization, using the method of permuted blocks with blocks of varying sizes and the uniform random number function in Stata version 16 and facilitated using REDCap, was used to assign caregivers to usual care or the intervention in a 1:1 ratio. Statistical analysis of data from both CRx trials follow the same general analytic plan (Supplement 1). The main analyses will evaluate outcomes by intent-to-treat [49].

Table 1 summarizes primary and secondary outcomes for both trials. Generalized linear mixed models (GLMM) will be utilized to leverage the longitudinal data, fit with a time by study arm interaction. The baseline value will be included as a covariate. Additionally, for both trials, all caregivers will be included in analyses to assess whether the intervention has an impact on caregiver satisfaction with care and experiences of discrimination. These data will be analyzed using a non-inferiority analysis. See Supplement 1 for additional details regarding measures administered, scoring, and statistical methods.

We attempt to reduce missing data by administering interviewer-administered surveys at baseline and providing ample opportunity and options for follow-up survey completion (e.g., self-completed online, phone-based survey completion with an interviewer, survey completion windows of up to a month for certain timepoints, etc.). Using these strategies, average item non-response ranges from 0 to 4% at each follow-up time point in the CommunityRx-Hunger and CommunityRx-Dementia trials. To avoid bias due to missing data (item non-response or dropout), multiple imputation, using the chained equations method, or inverse probability weighting will be employed. Due to the minimal risk of each study, no interim analyses were conducted and trial stopping rules did not apply.

### Dissemination

In addition to scientific publications and presentations, we have developed a dissemination plan in conjunction with our study CABs that includes dissemination to other communities of interest. This plan includes dissemination of study findings to various audiences through modes such as mail/email to study participants who give permission for contact, internal and external newsletters, and presentations to partner organizations. As we have done in prior studies [50], we plan to develop a report of findings to be published online and shared with all study participants.

## Discussion

Many clinical trials were immediately halted or encountered long delays as a result of the COVID-19 pandemic [51]. Published estimates show that only 40% of halted non-oncology trials had been reactivated as of March 2021 [52]. Despite documented challenges associated with caregiver-centered research, pandemic-related delays, and a clinical trial team working fully remotely, both of our trials were not only re-activated by late 2020 but yielded faster enrollment rates than projected pre-pandemic. The asset-based, community-engaged approach, combined with our innovation skills, enabled us to leverage strong, timely community advising and widely accessible institutional and commercial information technologies to facilitate rapid migration to remote trial operations. Our strategies and learnings can inform future social care interventional studies involving caregivers and other groups with limited access to traditional health services research participation.

For these two trials, rapid translation of in-person clinical trial protocols to a remote design was feasible due, in part, to a high-functioning network of stakeholders, community advisors, and environmental supports in place well before the COVID-19 crisis. During the pandemic, CABs for both trials met remotely to provide ongoing support and advocacy for continuing the research. In the case of CommunityRx-Hunger, CLS specialists on the CAB were especially critical to the successful iteration of the intervention delivery protocols. Of note for pediatric trialists, CLS programs operate in more than 400 pediatric hospitals, emergency departments, and community clinics in the USA [53], and engagement in research on the psychosocial needs of children and families are standards of their clinical practice [54]. In the case of CommunityRx-Dementia, Tech Savvy Friends was recommended by a CAB member during a tele-convening where remote protocols were being discussed. An introduction to Tech Savvy Friends enabled us to quickly incorporate this community resource into our enrollment protocols. Additionally, because these trials were identifying people with food insecurity—and rates were rising as a result of the pandemic [55, 56]—our ability to sustain and rapidly expand the Feed1st pantry program [31] was important to preserving the ethical conduct of research.

With contemporaneous funding for two large RCTs, we created the ARCTICS innovation before the pandemic to realize economies of scale and minimize caregiver burden. This innovative technology, along with rapid adoption of videoconferencing, became essential to sustaining the trials remotely [57]. In addition, based on guidance from CAB members, we added an ARCTICS-driven text message to our recruitment outreach strategy to increase the likelihood that our calls would be answered. This message let each caregiver know who we were and from which number we were calling before we initiated phone outreach. While consent rates during remote pre-testing were slightly lower than in-person for both studies, our remote recruitment strategies yielded higher approach rates than in-person protocols for both trials, allowing us to approach more people at a faster rate than in-person. Our remote recruitment strategies, informed by each study’s CAB, enabled us to accommodate caregivers’ schedules and recruit them at times and in ways that were most convenient for them.

For comparison, a prior cross-sectional study of household food insecurity among parents of children admitted to the same hospital consented 85% of eligible parents (versus 77% for the in-person pretest and 69% of the remote pretest for CommunityRx-Hunger) [32]. An intervention development study of decision-making experiences of people with dementia implemented similar videoconferencing and virtual consent procedures as described here to adapt to pandemic conditions [58]. Consent rate data for that study are not yet published. The demonstrated resilience of the ARCTICS innovation to pandemic conditions led to its adoption in other remotely-operated clinical trials, including the My Diabetes My Community (MDMC) Trial that launched in September 2021 with funding from the National Institutes of Diabetes, Digestive and Kidney Diseases [59]. Using ARCTICS, MDMC had remotely enrolled 74 of a planned 600 older patients with type 2 diabetes by December 2021.

Remote implementation of the CommunityRx studies also required major changes to the mode of intervention delivery that were not anticipated by the in-person design. In the remote scenario, the intervention could be delivered synchronously, meaning via videoconference during the child’s hospitalization (CommunityRx-Hunger) or soon after an outpatient clinical encounter (CommunityRx-Dementia), or asynchronously using text and email, as detailed above. Using implementation science methods, adaptations to the intervention were documented [60], and the provisional essential elements of each intervention are being systematically tracked to allow for a robust intervention fidelity assessment [61]. While not originally designed as pragmatic trials, iteration of the trials to adapt to external, real-world challenges is a hallmark of pragmatic trial design [62]. Our approach could be emulated by other trialists.

The CommunityRx trials introduce innovation to interventions informed by the Self- and Family Management Framework by expanding the ways and among whom the Framework is being used [11]. Specifically, our work adds to the growing number of RCTs using the Framework that included an e-health component, focused on dementia or health disparities, enrolled family caregivers, or have included a predominantly African American/Black sample [22]. The CommunityRx trials also highlight the relevance of the Self- and Family Management Framework in times of crisis. During the COVID-19 pandemic, the importance of the patient-family relationship came to the fore. Family members were largely unable to be present at the bedside where they would normally support patients, provide information to clinicians, and generally co-manage illness. The CommunityRx trials demonstrate a core concept of the Self- and Family Management Framework, which is the need to support caregiver needs related to family management (including family caregivers’ self-care) so that they can sustainably support patient self-management [63].

## Conclusions

In-person clinical trials enrolling caregivers of patients with severe or chronic illness face particular challenges, made only more apparent by the COVID-19 pandemic. Rapid iteration to a remote design of two social care RCTs was facilitated by longstanding community engagement and innovation to optimize trial efficiency using widely accessible institutional and commercial information technology tools. Beyond the pandemic, fully remote or hybrid RCT protocols for social care interventions may be a viable, scalable alternative to bedside protocols, including in studies of caregivers. These innovative design elements have implications for wider applicability and scalability for multi-site or adaptive trials or trials enrolling people with limited mobility or living in rural or other remote areas. This description of the methods and protocols deployed in concurrent caregiver-focused RCTs strengthens the evidence that the trial innovations are replicable and effective across interventions targeting different caregiver populations and facilitates comparative analysis of trial outcomes—an especially rare opportunity in the field of social care.

### Supplementary Information

**Additional file 1: Supplement 1. **Detailed statistical analysis plan for CommunityRx-Hunger and CommunityRx-Dementia.

**Additional file 2: Supplement 2. **Informed consent document used in CommunityRx-Hunger.

**Additional file 3: Supplement 3. **Informed consent document used for CommunityRx-Dementia.

## Data Availability

The datasets used and/or analyzed during the current study are available from the corresponding author on reasonable request.

## Abbreviations

API: Application programming interface
ARCTICS: Automated Randomized Controlled Trial Intervention-Communication System
CAB: Community Advisory Board
CLS: Child Life Services
EMR: Electronic medical record
HRSR: Health-related social risk
IRB: Institutional Review Board
RCT: Randomized controlled trial
