# Supplementary material for: CommunityRx, a social care assistance intervention for family and friend caregivers delivered at the point of care: two concurrent blinded randomized controlled trials
Source: Trials. 2023 Oct 21;24:681. doi: 10.1186/s13063-023-07697-z (PMC10624358; doi:10.1186/s13063-023-07697-z)
Supplement: Supplementary file 1 — Additional file 1: Supplement 1. Detailed statistical analysis plan for CommunityRx-Hunger and CommunityRx-Dementia. [file 13063_2023_7697_MOESM1_ESM.docx]

**Supplement 1.** Detailed statistical analysis plan for CommunityRx-Hunger and CommunityRx-Dementia

Table 1 in the main narrative describes the design characteristics, scientific aims and corresponding outcomes of CommunityRx-Hunger and CommunityRx-Dementia.

Aim 1 analyses will evaluate the impact of the intervention on self-efficacy, limiting the analytic sample to food insecure caregivers for CommunityRx-Hunger and caregivers with at least 1 HRSR for CommunityRx-Dementia. For CommunityRx-Hunger, the primary outcome of caregiver self-efficacy for finding resources is assessed using an adapted measure of Bandura’s 1-item Self-Efficacy Scale,(1,2) “How confident are you in your ability to find resources in your community that help you manage your health?” Responses will be assessed on a 1 (“not at all confident”) to 5-point (“completely confident”) Likert scale. Self-efficacy is queried at baseline, 7 days, 30 days, 90 days, 6 months, and 12 months. Secondary outcomes include severity of household food insecurity,(3) caregiver health, assessed using the Medical Outcomes Study Short Form-12,(4) caregiver-reported child health, using the single-item physical and mental health measure from the National Survey of Child Health,(5) adult and child nutrition, and child healthcare utilization obtained from the child’s electronic medical record and caregiver self-report. For the primary outcome of self-efficacy for finding resources at 12-months, 236 food insecure caregivers per arm allows detection of a treatment difference of a similar magnitude as that from a previous CommunityRx trial(6) (3.56 +/- 1.47 vs. 3.92 +/- 1.24). These calculations, performed during the study design phase, assumed a group allocation of 1:1, power=0.8, alpha=0.05, and incorporated a nonparametric adjustment.

For CommunityRx-Dementia, caregiver self-efficacy is assessed using the 4-item self-efficacy sub-domain from the 2015 Caregiver Dementia Care and Self-Efficacy Survey(7) that utilizes a 1 (“not at all confident”) to 5-point (“completely confident”) Likert scale. Per protocol, responses will be averaged. Self-efficacy is queried at baseline, 30 days, 90 days and 12 months. Secondary outcomes include psychosocial outcomes (unmet needs,(8) social isolation, caregiver well-being, caregiver burden,(9) depression,(10) and stress(11)), behavioral outcomes (use of the web-based Community Resource Finder), and health and healthcare outcomes (caregiver physical health and quality of life,(12) physical health of the care recipient,(4) and healthcare utilization). For the primary outcome of caregiver self-efficacy at 12 months, 86 caregivers with unmet HRSRs in each arm allows for a detectable treatment difference of 0.43 points, a minimally important difference. Calculations assumed a group allocation of 1:1, power=0.8, alpha=0.05, mean=2.8 in the control group, and SD=1.

Generalized linear mixed models (GLMM) will be utilized to leverage the longitudinal data, fit with a time by study arm interaction. Time points will include 7, 30, 90, 180 days and 12 months post-discharge for CommunityRx-Hunger and 7, 30, 90 days and 12 months for CommunityRx-Dementia. The baseline value will be included as a covariate.

Aim 2 analyses include all randomized caregivers and will assess whether the intervention has an impact on caregiver satisfaction with care (measured at 7 days) and experiences of discrimination. For CommunityRx-Hunger, the primary outcome of interest is assessed using the Child Hospital Consumer Assessment of Healthcare Providers Survey (HCAHPS),(13) which queries satisfaction with discharge. The total score will be transformed to a 0-100 scale for ease of interpretation. Assuming group allocation of 1:1, one-sided alpha=0.05, standard deviation of 8, and a non-inferiority margin of 1.6, a total sample size of 640 is needed to achieve 80% power. For CommunityRx-Dementia, the primary outcome of satisfaction with care is assessed using the Patient Satisfaction Questionnaire 18-item short form (PSQ-18).(14) With a total sample size of 344, there would be 80% power (one-sided alpha=0.025, SD=25) to detect a non-inferiority margin of 7.6. In both trials, discrimination is assessed using the Discrimination in Medical Settings Scale (DMS).(15) Non-inferiority will be supported for the primary outcome if the upper limit of the confidence interval for the treatment difference (usual care – CommunityRx-Hunger or CommunityRx-Dementia) does not exceed the non-inferiority margin.

Aims 1 and 2 for each trial each have one primary outcome. Sample size calculations for each trial did not account for multiple primary outcomes because, for both trials, Aim 2 utilizes data from the full trial whereas Aim 1 only includes a subsample of the full sample.

**REFERENCES**

1. Bandura A. Self-efficacy: toward a unifying theory of behavioral change. Psychol Rev. 1977;84(2):191.

2. Bandura A. Guide for constructing self-efficacy scales. In: Self-Efficacy Beliefs of Adolescents [Internet]. Information Age Publishing; 2006 [cited 2018 Feb 23]. p. 307–37. Available from: https://www.uky.edu/~eushe2/Bandura/BanduraGuide2006.pdf

3. The Accountable Health Communities Health-Related Social Needs Screening Tool [Internet]. Center for Medicare & Medicaid Services. [cited 2021 Jul 7]. Available from: https://innovation.cms.gov/Files/worksheets/ahcm-screeningtool.pdf

4. Ware JE, Kosinski M, Bayliss MS, McHorney CA, Rogers WH, Raczek A. Comparison of methods for the scoring and statistical analysis of SF-36 health profile and summary measures: summary of results from the Medical Outcomes Study. Med Care. 1995 Apr;33(4 Suppl):AS264-279.

5. National Survey of Children’s Health, 2003 [Internet]. Health Resources and Services Administration, Maternal and Child Health Bureau, Centers for Disease Control and Prevention, National Center for Health Statistics; 2003 [cited 2016 Feb 29]. Available from: http://www.cdc.gov/nchs/data/slaits/nsch_questionnaire.pdf

6. Lindau ST, Makelarski J, Abramsohn E, Beiser DG, Boyd K, Chou C, et al. CommunityRx: A real-world controlled clinical trial of a scalable, low-intensity community resource referral intervention. Am J Public Health. 2019 Apr;109(4):600–6.

7. Steffen AM, McKibbin C, Zeiss AM, Gallagher-Thompson D, Bandura A. The revised scale for caregiving self-efficacy: reliability and validity studies. J Gerontol B Psychol Sci Soc Sci. 2002 Jan;57(1):P74-86.

8. Jennings LA, Reuben DB, Evertson LC, Serrano KS, Ercoli L, Grill J, et al. Unmet needs of caregivers of individuals referred to a dementia care program. J Am Geriatr Soc. 2015 Feb;63(2):282–9.

9. Peipert JD, Jennings LA, Hays RD, Wenger NS, Keeler E, Reuben DB. A composite measure of caregiver burden in dementia: The Dementia Burden Scale-Caregiver. J Am Geriatr Soc. 2018 Sep;66(9):1785–9.

10. Arroll B, Goodyear-Smith F, Crengle S, Gunn J, Kerse N, Fishman T, et al. Validation of PHQ-2 and PHQ-9 to screen for major depression in the primary care population. Ann Fam Med. 2010 Jul 1;8(4):348–53.

11. Cohen S, Kamarck T, Mermelstein R. A global measure of perceived stress. J Health Soc Behav. 1983 Dec;24(4):385–96.

12. Salsman JM, Victorson D, Choi SW, Peterman AH, Heinemann AW, Nowinski C, et al. Development and validation of the positive affect and well-being scale for the neurology quality of life (Neuro-QOL) measurement system. Qual Life Res Int J Qual Life Asp Treat Care Rehabil. 2013 Nov;22(9):2569–80.

13. Goldstein E, Elliott MN, Lehrman WG, Hambarsoomian K, Giordano LA. Racial/ethnic differences in patients’ perceptions of inpatient care using the HCAHPS survey. Med Care Res Rev MCRR. 2010 Feb;67(1):74–92.

14. Marshall GN, Hays RD. The Patient Satisfaction Questionnaire Short-Form (PSQ-18) [Internet]. RAND. 1994 [cited 2018 Dec 13]. Available from: https://www.rand.org/content/dam/rand/pubs/papers/2006/P7865.pdf

15. Peek ME, Nunez-Smith M, Drum M, Lewis TT. Adapting the everyday discrimination scale to medical settings: Reliability and validity testing in a sample of African American patients. Ethn Dis. 2011;21(4):502–9.
