# Supplementary material for: CommunityRx, a social care assistance intervention for family and friend caregivers delivered at the point of care: two concurrent blinded randomized controlled trials
Source: Trials. 2023 Oct 21;24:681. doi: 10.1186/s13063-023-07697-z (PMC10624358; doi:10.1186/s13063-023-07697-z)
Supplement: Supplementary file 2 — Additional file 2: Supplement 2. Informed consent document used in CommunityRx-Hunger. [file 13063_2023_7697_MOESM2_ESM.pdf]

Supplement 2. Informed consent document used in CommunityRx-Hunger

The UNIVERSITY OF CHICAGO
The Division of the Biological Sciences • The University of Chicago Medical Center

CONSENT/AUTHORIZATION FOR PARTICIPATION IN A RESEARCH PROTOCOL

Protocol Number: IRB20-0324 Name of Subject: ____________________________

STUDY TITLE: CommunityRx

Doctors Directing Research: Stacy Tessler Lindau, MD, MAPP
Address: University of Chicago, 5841 S. Maryland Ave. MC 2050, Chicago, IL 60637
Telephone Number: (773) 834-8986

Study Coordinator: Emily Abramsohn, MPH
Address: University of Chicago, 5841 S. Maryland Ave. MC 2050, Chicago, IL 60637
Telephone Number: (773) 834-4832

KEY INFORMATION

We are asking you to choose whether or not to volunteer for a research study about food security and related needs among parents and other caregivers of hospitalized children or children with a serious illness. Food security means the availability of food and a person's ability to access it. The purpose of this study is to help improve adult and child health and narrow gaps in health between lower income and higher income families by addressing food security and related needs.

We are studying two different ways to give families information about food and other resources during and after a child is discharged from the hospital or their day treatment appointment. We want to learn if one way is better than the other. You will be randomly assigned to one of two groups. You have an equal chance of being assigned to either group, but you will not know which group you are assigned to.

This section is to give you key information to help you decide whether to participate. We have included detailed information after this page. Ask the research team questions. If you have questions later, contact the research investigator in charge of the study above.

WHAT IS THE STUDY ABOUT AND HOW LONG WILL IT LAST?

By doing this study, we hope to learn how best to support the food and other resource needs of parents and other caregivers of children during and after discharge from the hospital or their day treatment appointment. Your participation in this research will last about one year. You will be asked to take a survey during your child's hospitalization or their day treatment appointment

and again by phone or online in one week, one month, 3 months, six months and one year. You will also receive text messages from the research team during this time.

In addition to your consent for your participation in this research, we are asking to look at some information in your child's medical and billing records. We would need your permission for this. The only information we will get from your child's medical record is 1) the number of times your child goes to the doctor, 2) the dates of those doctor visits, 3) the dates of any hospital stays your child may have in the next year, 4) the dates of any visits to the emergency department for your child in the next year, and 5) any charges associated with those visits. Your child would not have to answer any questions or provide any other information. **You are still able to participate in this research even if you do not provide permission to access your child's medical record.** You do not need to decide now; we can talk about it again when we follow up with you.

WHAT ARE KEY REASONS YOU MIGHT CHOOSE TO VOLUNTEER FOR THIS STUDY?
While there is no direct benefit for participating in this study, there may be a benefit to society by contributing to research to support parents and other caregivers of children during and after being discharged from the hospital or their day treatment appointment. For a complete description of benefits, refer to the Detailed Consent.

WHAT ARE KEY REASONS YOU MIGHT CHOOSE NOT TO VOLUNTEER FOR THIS STUDY?
The main reason why you might not choose to volunteer for this study is the risk of loss of confidentiality (privacy). This risk is very low, and we will do everything we can to minimize this risk. The alternative to volunteering in this study is not to participate. For a complete description of risks, refer to the Detailed Consent.

DO YOU HAVE TO TAKE PART IN THE STUDY?
Taking part in this study is voluntary. If you decide to take part in the study, it should be because you really want to volunteer. You will not lose any services, benefits or rights you would normally have if you choose not to volunteer.

You may choose not to participate at any time during the study. You will not lose any services, benefits or rights you would normally have if you choose to leave the study. The University of Chicago/University of Chicago Medical Center will not condition (withhold or refuse) treating you on whether you sign this Authorization or revoke your authorization at a later time. If you do not sign this form, you will not receive the research-related intervention(s).

WHAT IF YOU HAVE QUESTIONS, SUGGESTIONS OR CONCERNS?
The person in charge of the study is Stacy Tessler Lindau, MD, MAPP of the University of Chicago. If you have questions, suggestions, or concerns regarding this study or you want to withdraw from the study her contact information, by phone, is: (773) 834-8986.

For questions about your rights as a research subject, please contact the University of Chicago BSD IRB at 773-702-6505.

OTHER KEY INFORMATION
We also want to tell you that some of the information tools we are using for this study are provided by UniteUs, LLC. Dr. Lindau, who is the principal investigator on this study, founded a company called NowPow which developed some of the information tools being used for this study. This company has now been acquired by a company called UniteUs, and Dr. Lindau owns equity in this company and is a paid advisor to UniteUs. Both Dr. Lindau and the University of Chicago do stand to potentially receive financial benefit from this company if these tools prove to be successful. If you have any questions about this information, you can call Dr. Lindau. Her phone number is (773) 834-8986.

DETAILED CONSENT

WHAT IS MY INVOLVEMENT IN THE STUDY?

About 640 caregivers and their children will take part in this study at the University of Chicago.

- This research is studying two different ways to give families information about food and other resources during and after a child is discharged from the hospital or a day treatment appointment. We want to learn if one way is better than the other. Other resources could include where to get medication for a child and who to contact with questions or for help.

- You will be randomly assigned to one of two groups. You have an equal chance of being assigned to each group, but you will not know which group you are assigned to.

- During this study, you will receive one or more visits, either in person or by phone or video, by a person from our research team before your child is discharged from the hospital or their day treatment appointment.

- Around the time your child is discharged from the hospital or their day treatment appointment, you will receive a text message from our research team. You will have the opportunity to receive messages from them and send messages to them throughout the study.

- During this study, Dr. Lindau and her research team will collect information from you at 6 different times: today and again in one week, one month, three months, 6 months and one year. We will collect this information either by phone or through a web-based survey.

- If your child has a longer than anticipated hospital stay, you may become ineligible for future follow up surveys. If this happens, we will contact you to let you know and we will stop calling you and sending you text messages.

- In the future, identifiers associated with your data could be removed from the data. The de-identified data could then be used for future research by our research team or other researchers without notifying you or asking your permission for this use.

WHAT IS MY CHILD'S INVOLVEMENT IN THIS STUDY?

In addition to your consent for your participation in this research, we are asking to look at some information in your child's medical and billing records. Again, we are studying two different ways to give families information, and we want to learn if they have a different effect on recommended follow-up visits. We would need your permission for this. In addition to your child's name and medical record number, the only information we will get from your child's medical record is:

1) the number of times your child goes to the doctor,
2) the dates of those doctor visits,
3) the dates of any hospital stays your child may have in the next year,
4) the dates of any visits to the emergency department for your child, and
5) any charges associated with those visits.

We will view and collect these data from your child's electronic medical record and billing records and will only collect data pertaining to the year following their discharge from the hospital or their day treatment appointment. We will collect these data within one year of the end of the study.

We will not collect at any other information in your child's medical record. Collecting information other than what is described above would not be allowed without your permission.

If your child is 7 years old or older, we will also ask their permission through a similar process called "assent." If your child is younger than 7, their assent is waived, meaning we would only need your permission. Your child will not be asked to answer any questions or provide any other information. **You are still able to participate in this research even if you do not provide permission to access your child's medical record.** You can make this decision now by signing your name at the bottom of this form where we ask for your permission. Or, you can take some time to think about allowing us to collect these data and decide later. We can talk about it further when we follow up with you next.

WHAT ARE THE RISKS OF THE STUDY?
This study has minimal risks. There is a risk of loss of confidentiality (privacy); this risk is very unlikely and we will do everything we can to minimize those risks.

Psychological and/or emotional discomfort associated with the survey questions is possible, but this is also very unlikely. You can refuse to answer any question and your participation in the research is voluntary, meaning it is completely up to you whether you participate or not.

We are required by law to report to the local authorities any incidents of abuse or neglect of children or vulnerable adults that we may learn about while conducting the research. This report would include your name, telephone number or address and the reason why we suspect abuse or neglect.

If during the course of the study we believe you are in immediate health danger, we will call 911. When the paramedics arrive, you can refuse treatment if you do not want to go to the hospital. If you choose to go to the hospital, the researchers are not responsible for any costs that you may have from going to the hospital.

ARE THERE ANY BENEFITS TO TAKING PART IN THE STUDY?
Being in this study may not help you directly. We hope that your participation in the study may benefit other people in the future by helping us learn more about how best to support

caregivers of hospitalized children.

WHAT OTHER OPTIONS ARE THERE?

Instead of being in this study, you may choose not to participate. If you do not want to participate but would like some of the information provided in the study, we would be happy to provide you with this information.

WHAT ARE THE COSTS?

There will be no costs to you or your insurance company for taking part in this research study. However, you or your insurance company will be responsible for costs related to your usual medical care.

WHAT HAPPENS IF I HAVE AN INJURY?

There will be no costs to you or your insurance company resulting from your participation in this research study. However, you or your insurance company will be responsible for costs related to your usual medical care.

WILL I BE PAID FOR MY PARTICIPATION?

For participation in this study, you will receive a $20 gift card of our choice in compensation for completion of the baseline survey (~25 minutes). You will receive a $25 Visa gift card in compensation upon completion of each of the follow up surveys (~30 minutes) for a total of up to $145. You can choose to receive your compensation by email, which you will receive within 5-7 days or by mail which you will receive within 4 weeks of completing each of the surveys.

WHAT ABOUT MY CONFIDENTIALITY?

We will collect information from you during the course of the study that could identify you personally. Any study records that identify you will be kept confidential. Specifically, we will collect your name, telephone number, address and email address and certain dates (like your birthdate and the date of medical services). This information is referred to as Protected Health Information (PHI). PHI consists of any health information that is collected about you, which could include your medical history and new information collected as a result of this study. We will keep PHI in order to follow-up with you for future surveys and pay you for your participation. We will either de-identify your responses, meaning that we will remove any information that may identify you, or limit your PHI to the minimum amount necessary to analyze your responses for this study.

We will collect your name, telephone number and date of enrollment into the study to conduct the follow-up surveys. This information will be sent to a text messaging service called Mosio so we can send you text messages about the study. Your name, telephone number, address and date of birth may also be sent to an information tool called NowPow. If so, this information will be kept by Mosio and NowPow only for the duration of the study. When the study is over, both Mosio and NowPow will destroy this information. Both Mosio and NowPow meet University of Chicago Medicine’s security standards.

We will collect your name and address in order to pay you for your participation; this information will be given to our accounting department for reconciliation of your payment. We will also ask for your permission to contact you for future research; this will be documented on this consent form. If you say yes, we will collect your name, telephone number, address and email address. This information will in no way be linked to your responses. All information we collect will be entered into a password-protected database on password-protected computers. All paper copies of the study materials will be kept in locked file cabinets in locked offices at the University of Chicago; any electronic study materials will be stored on password-protected computers. Access to PHI collected for recruitment, data collection, payment or to re-contact you, is strictly limited to research staff and stored on secure, password-protected computers in locked offices. Backup files of secure data will be stored in locked file cabinets in locked rooms. All researchers working with this information, including the people collecting and accessing secure information, go through ethics training about conducting research with human subjects.

Participants in this study will not be identified in any report, publication, or presentation of this study or its results. Information will be used for studying of groups of people, never for individuals. No one will know that you took part in this study unless you tell them.

Your records may be reviewed by federal agencies whose responsibility is to protect human subjects in research including the Office of Human Research Protections (OHRP). In addition, representatives from the University of Chicago, including the Institutional Review Board, a committee that oversees the research at the University of Chicago, may also view the records of the research. If your research record is reviewed by any of these groups, they may also need to review your entire medical record.

This consent form will be kept by the research team for at least six years. The study results will be kept in your research record and be used by the research team indefinitely. Information from this study may be used in medical publications or presentations. Again, your name and any other information that could identify you will be removed before these data are presented.

At the time of study completion, either the research information will be destroyed or information identifying you will be removed from study results.

A description of this clinical trial will be available on http://www.ClinicalTrials.gov, as required by U.S. Law. This Web site will not include information that can identify you. At most, the Web site will include a summary of the results. You can search this Web site at any time.

To help us protect your privacy, the National Institutes of Health (NIH) has issued a Certificate of Confidentiality for this research. With this Certificate, the researchers cannot be forced to disclose information that may identify you, even by a court subpoena, in any federal, state, or local civil, criminal, administrative, legislative, or other proceedings. The researchers will use the Certificate to resist any demands for information that would identify you, except as explained below. The Certificate cannot be used to resist a demand for information from personnel of the United States Government that is used for auditing or evaluation of federally

funded projects or for information that must be disclosed in order to meet the requirements of the federal Food and Drug Administration (FDA).

A Certificate of Confidentiality does not prevent you or a member of your family from voluntarily releasing information about yourself or your involvement in this research. If an insurer, employer, or other person obtains your written consent to receive research information, then the researchers may not use the Certificate to withhold that information. There are specific circumstances when the Certificate of Confidentiality does not prevent researchers from disclosing voluntarily, without your consent, information that would identify you as a participant in the research. For example: suspected child abuse, elder abuse, or urgent risk of harm to self (suicide) or others (homicide).

WHAT ABOUT MY CHILD'S CONFIDENTIALITY?
We will access your child's name and medical record number in order to access his or her electronic medical record. We will access your child's health insurance identifier to obtain their health insurance claims information. We will keep your child's PHI only to ensure that we are accessing the correct medical or billing records. When we analyze the data, we will either de-identify the data, meaning that we will remove any information that may identify them, or limit their PHI to the minimum amount necessary. More information about the confidentiality of your child's information is included in their assent form.

WHAT ARE MY RIGHTS AS A PARTICIPANT?
If you choose to no longer be in the study and you do not want any of your future health information to be used, you must inform Dr. Stacy Lindau in writing at the address on the first page. Dr. Stacy Lindau may still use your information that was collected prior to your written notice.

You will be given a signed copy of this document. Your authorization to use and disclose your health information does not have an expiration date.

MAY WE CONTACT YOU IN THE FUTURE?
Lastly, we would like to be able to contact you in the future in the event that there are other studies that you may be interested in or eligible for. We will keep your name, telephone number, address and email address in order to re-contact you. This portion is optional; if you do not agree to be re-contacted, it does not affect your ability to be in the current study.

__ Yes, I agree to be re-contacted for future research
__ No, I do not agree to be re-contacted for future research

Initials of Subject: ____________________________
Date: ___________

CONSENT FOR MY PARTICIPATION (For participants ages 18 and older):

SUBJECT
The research project and the procedures associated with it have been explained to me. The experimental procedures have been identified and no guarantee has been given about the possible results. I will receive a signed copy of this consent form for my records.

I agree to participate in this study. My participation is voluntary and I do not have to sign this form if I do not want to be part of this research study.

Signature of Subject: ______________________________
Date: ___________ Time: ______ AM/PM (Circle)

ASSENT FOR MY PARTICIPATION (For participants between the ages of 16-17):

SUBJECT
The research project and the procedures associated with it have been explained to me. The experimental procedures have been identified and no guarantee has been given about the possible results. I will receive a signed copy of this assent form for my records.

I agree to participate in this study. My participation is voluntary and I do not have to sign this form if I do not want to be part of this research study.

Signature of Subject: ______________________________
Date: ___________ Time: ___________ AM/PM (Circle

PERSON OBTAINING CONSENT
I have explained to ______________________________ the nature and purpose of the study and the risks involved. I have answered and will answer all questions to the best of my ability. I will give a signed copy of the consent form to the subject.

Signature of Person Obtaining Consent: __________________________
Date: ___________ Time: ___________ AM/PM (Circle)

PERMISSION FOR MY CHILD'S PARTICIPATION:

PARENT/GUARDIAN/ OR LEGALLY AUTHORIZED REPRESENTATIVE:
I give my permission for my child/relative/the person I represent to participate in the above described research project.

Signature of Parent/Guardian/ or Legally Authorized Representative: ___________________
Date: ___________ Time: ___________ AM/PM (Circle)
