# Supplementary material for: CommunityRx, a social care assistance intervention for family and friend caregivers delivered at the point of care: two concurrent blinded randomized controlled trials
Source: Trials. 2023 Oct 21;24:681. doi: 10.1186/s13063-023-07697-z (PMC10624358; doi:10.1186/s13063-023-07697-z)
Supplement: Supplementary file 3 — Additional file 3: Supplement 3. Informed consent document used for CommunityRx-Dementia. [file 13063_2023_7697_MOESM3_ESM.pdf]

Supplement 3. Informed consent document used for CommunityRx-Dementia

The UNIVERSITY OF CHICAGO
The Division of the Biological Sciences • The University of Chicago Medical Center

CONSENT/AUTHORIZATION FOR PARTICIPATION IN A RESEARCH PROTOCOL

Protocol Number: IRB20-0301 Name of Subject: ___________________

STUDY TITLE: CommunityRx-Caregiver

Doctors Directing Research:
Stacy Tessler Lindau, MD, MAPP
Address: University of Chicago, 5841 S. Maryland Ave. MC 2050, Chicago, IL 60637
Telephone number: (773) 834-8986

Elbert Huang, MD, MPH
Address: University of Chicago, 950 E. 59th St., AMB B214, Chicago, IL 60637
Telephone number: 773-834-3419

Study Coordinator:
Jyotsna S. Jagai, MS, MPH, PhD
Address: University of Chicago, 5841 S. Maryland Ave. MC 2050, Chicago, IL 60637
Telephone Number: (773) 834-8534

KEY INFORMATION

We are asking you to choose whether or not to volunteer for a research study about unmet needs among caregivers of people with Alzheimer's disease and other dementias. Unmet needs could include things like food, housing, and social support. The purpose of this study is to help improve the health of caregivers and people with dementia and narrow gaps in health by addressing unmet health and social needs. We are studying two different ways to give caregivers information about health and social needs during a clinical visit. We want to learn if one way is better than the other. You will be randomly assigned to one of two groups. You have an equal chance of being assigned to either group, but you will not know which group you are assigned to. This section gives you key information to help you decide whether to participate. We have included detailed information after this page. Ask the research team questions. If you have questions later, contact anyone on the research team listed above.

WHAT IS THE STUDY ABOUT AND HOW LONG WILL IT LAST?

By doing this study, we hope to learn how best to support the needs of caregivers of people with dementia, starting at the point of their own healthcare. Your participation in this research will last about one year. You will be asked to take a survey today and again by phone or online in one week, one month, 3 months, and 12 months. You will also receive text messages and

emails from the research team during this time. With your permission, we will also view and collect data from your electronic medical record and health insurance claims.

WHAT ARE KEY REASONS YOU MIGHT CHOOSE TO VOLUNTEER FOR THIS STUDY?
While there is no direct benefit for participating in this study, there may be a benefit to society by contributing to research to support caregivers of people with dementia. For a complete description of benefits, refer to the Detailed Consent.

WHAT ARE KEY REASONS YOU MIGHT CHOOSE NOT TO VOLUNTEER FOR THIS STUDY?
The main reason why you might not choose to volunteer for this study is the risk of loss of confidentiality (privacy). This risk is very low, and we will do everything we can to minimize this risk. The other reason you might not choose to volunteer for this study is the risk of psychological and/or emotional discomfort associated with the survey questions. You can refuse to answer any questions and your participation in the research is voluntary. The alternative to volunteering in this study is not to participate. For a complete description of risks, refer to the Detailed Consent.

DO YOU HAVE TO TAKE PART IN THE STUDY?
Taking part in this study is voluntary. If you decide to take part in the study, it should be because you really want to volunteer. You will not lose any services, benefits or rights you would normally have if you choose not to volunteer.

You may choose not to participate at any time during the study. You will not lose any services, benefits or rights you would normally have if you choose to leave the study. The University of Chicago/University of Chicago Medical Center will not condition (withhold or refuse) treating you on whether you sign this Authorization or revoke your authorization at a later time. If you do not sign this form, you will not receive the research-related intervention(s).

WHAT IF YOU HAVE QUESTIONS, SUGGESTIONS OR CONCERNS?
The people in charge of the study are Stacy Tessler Lindau, MD, MAPP and Elbert Huang, MD, MPH of the University of Chicago. If you have questions, suggestions, or concerns regarding this study contact Dr. Lindau or Dr. Huang. Dr. Lindau's phone number is: (773) 834-8986. Dr. Huang's phone number is: (773) 834-9143.

For questions about your rights as a research subject, please contact the University of Chicago Biological Sciences Division Institutional Review Board at 773-702-6505.

OTHER KEY INFORMATION
We also want to tell you that some of the information tools we are using for this study are provided by UniteUs, LLC. Dr. Lindau, who is the principal investigator on this study, founded a company called NowPow which developed some of the information tools being used for this study. This company has now been acquired by a company called UniteUs, and Dr. Lindau owns equity in this company and is a paid advisor to UniteUs. Both Dr. Lindau and the

University of Chicago do stand to potentially receive financial benefit from this company if these tools prove to be successful. If you have any questions about this information, you can call Dr. Lindau. Her phone number is (773) 834-8986.

DETAILED CONSENT

WHAT IS INVOLVED IN THE STUDY?

About 344 caregivers of people with dementia will take part in this study at the University of Chicago.

- This research is studying two different ways to give caregivers information about health and social needs. We want to learn if one way is better than the other. Information about health and social needs could include where to get medication and who to contact with questions or for help.

- You will be randomly assigned to one of two groups. You have an equal chance of being assigned to each group, but you will not know which group you are assigned to.

- We will be connecting with you after the health appointment.

- After the visit, you will get a text message from us. You will have the opportunity to receive messages from us and send messages to us throughout the study.

- By signing this form, you also give us permission to look at and collect information from your electronic medical record and your health insurance claims. Again, we are studying two different ways to give caregivers information, and we want to learn if they have a different effect on caregiver's use of healthcare. We would only collect data pertaining to the time you are in the study and collect this data within one year of the end of the study. The only information we would get from your medical record and health insurance claims are:

    1. The number of times you go to the doctor, have a hospital stay or go to the emergency room;
    2. The dates of those visits;
    3. Any charges associated with those visits; and
    4. Whether any of your doctors noted that you were providing care to a person with dementia.

    We will not look at any other information in your medical record.

- During this study, Dr. Lindau and her research team will collect information from you at 7 different times. We will survey you today and contact you for another survey in one week, 1 month, 3 months, and 12 months. We will also contact you in 6 and 9 months to check that your contact information is up-to-date. The survey today will be run by a research member on Dr. Lindau's team here at the University of Chicago. Throughout this study, the researchers will collect information from you and other caregivers and ask you questions about the health of the person you care for that will help us with this research.

- In the future, identifiers associated with your data could be removed from the data. The de-identified data could then be used for future research by our research team or other researchers without notifying you or asking your permission for this use.

WHAT ARE THE RISKS OF THE STUDY?
This study has minimal risks. There is a risk of loss of confidentiality (privacy); this risk is very unlikely and we will do everything we can to minimize those risks.

Psychological and/or emotional discomfort associated with the survey questions is possible, but this is also very unlikely. You can refuse to answer any question and your participation in the research is voluntary, meaning it is completely up to you whether you participate or not.

We are required by law to report to the local authorities any incidents of abuse or neglect of children or vulnerable adults that we may learn about while conducting the research. This report would include your name, telephone number or address and the reason why we suspect abuse or neglect.

If during the course of the study we believe you are in immediate health danger, we will call 911. When the paramedics arrive, you can refuse treatment if you do not want to go to the hospital. If you choose to go to the hospital, the researchers are not responsible for any costs that you may have from going to the hospital.

ARE THERE ANY BENEFITS TO TAKING PART IN THE STUDY?
Being in this study may not help you directly. We hope that your participation in the study may benefit other people in the future by helping us learn more about how best to support caregivers of people with dementia.

WHAT OTHER OPTIONS ARE THERE?
Instead of being in this study, you may choose not to participate. If you do not want to participate but would like some of the information provided in the study, we would be happy to provide you with this information.

WHAT ARE THE COSTS?
There will be no costs to you or your insurance company for taking part in this research study. However, you or your insurance company will be responsible for costs related to your usual medical care.

WILL I BE PAID FOR MY PARTICIPATION?
For participation in this study, you will receive a $20 gift card in compensation for completion of the survey today (about 30 minutes). You will receive a $20 gift card in compensation for completion of the 1-week and 1-month survey (about 30 minutes each). You will receive a $30 gift card in compensation for completion of the 3-month survey (about 40 minutes) and a $50 gift card in compensation for completion of the 12-month survey (about 50 minutes). In total,

you may be compensated for a total of up to $140. You can choose to receive your compensation electronically by email within 5-7 days of completing each survey or by mail within 4 weeks of completing each of the surveys.

You will be entered into a raffle to win a $50 gift card if you verify your contact information is up-to-date when we contact you in 6-months and in 9-months. Each verification (one at 6-months and one at 9-months) counts as one raffle entry. Within 3 months of your check-in, you will receive an email notification about whether you are the raffle winner. If you're the winner, you can choose to receive your compensation electronically by email within 5-7 days of the email notification or by mail within 4 weeks of the email notification.

WHAT ABOUT CONFIDENTIALITY?

Study records that identify you will be kept confidential. We will collect information from you during the course of the study that could identify you personally; specifically, we will collect your name, telephone number, address, email address, certain dates (like your birthdate and the dates of medical services) and your medical record number. This information is referred to as Protected Health Information (PHI). PHI consists of any health information that is collected about you, which could include your medical history and new information collected as a result of this study. We will keep PHI in order to follow-up with you for future surveys and pay you for your participation. We will either de-identify your responses, meaning that we will remove any information that may identify you, or limit your PHI to the minimum amount necessary to analyze your responses for this study.

We will collect your name and telephone number into the study to conduct the follow-up surveys. This information will be sent to a text messaging service called Mosio so we can send you text messages about the study. Your name, address and date of birth may also be sent to an information tool called NowPow. If so, this information will be kept by Mosio and NowPow only for the duration of this study. When the study is over, both Mosio and NowPow will destroy this information. Both Mosio and NowPow meet University of Chicago Medicine's security standards.

We will collect your name and address in order to pay you for your participation; this information will be given to our accounting department for reconciliation of your payment. We will use your medical record number to access your electronic medical record. We will access your health insurance identifier to obtain health insurance claims information. We will also ask for your permission to contact you for future research; this will be documented on this consent form. If you say yes, we will collect your name, telephone number, address and email address. This information will in no way be linked to your responses. All information we collect will be entered into a password-protected database on password-protected computers. All paper copies of the study materials will be kept in locked file cabinets in locked offices at the University of Chicago; any electronic study materials will be stored on password-protected computers. Access to PHI collected for this research is strictly limited to approved research staff and stored on secure, password-protected computers in locked offices. Backup files of secure data will be stored in locked file cabinets in locked rooms. All researchers working with

this information, including the people collecting and accessing secure information, go through ethics training about conducting research with human subjects.

Participants in this study will not be identified in any report, publication, or presentation of this study or its results. Information will be used for studying of groups of people, never for individuals. No one will know that you took part in this study unless you tell them.

Your records may be reviewed by federal agencies whose responsibility is to protect human subjects in research including the Office of Human Research Protections (OHRP). In addition, representatives from the University of Chicago, including the Institutional Review Board, a committee that oversees the research at the University of Chicago, may also view the records of the research. If your research record is reviewed by any of these groups, they may also need to review your entire medical record.

This consent form will be kept by the research team for at least six years. The study results will be kept in your research record and be used by the research team indefinitely. Information from this study may be used in medical publications or presentations. Again, your name and any other information that could identify you will be removed before these data are presented. At the time of study completion, either the research information will be destroyed or information identifying you will be removed from study results.

A description of this clinical trial (NCT04146545) will be available on http://www.ClinicalTrials.gov, as required by U.S. Law. This Web site will not include information that can identify you. At most, the Web site will include a summary of the results. You can search this Web site at any time.

To help us protect your privacy, the National Institutes of Health (NIH) has issued a Certificate of Confidentiality for this research. With this Certificate, the researchers cannot be forced to disclose information that may identify you, even by a court subpoena, in any federal, state, or local civil, criminal, administrative, legislative, or other proceedings. The researchers will use the Certificate to resist any demands for information that would identify you, except as explained below. The Certificate cannot be used to resist a demand for information from personnel of the United States Government that is used for auditing or evaluation of federally funded projects or for information that must be disclosed in order to meet the requirements of the federal Food and Drug Administration (FDA).

A Certificate of Confidentiality does not prevent you or a member of your family from voluntarily releasing information about yourself or your involvement in this research. If an insurer, employer, or other person obtains your written consent to receive research information, then the researchers may not use the Certificate to withhold that information. There are specific circumstances when the Certificate of Confidentiality does not prevent researchers from disclosing voluntarily, without your consent, information that would identify you as a participant in the research. For example: suspected child abuse, elder abuse, or urgent risk of harm to self (suicide) or others (homicide).

WHAT ARE MY RIGHTS AS A PARTICIPANT?
If you choose to no longer be in the study and you do not want any of your future health information to be used, you must inform Dr. Stacy Lindau or Dr. Elbert Huang in writing and mail your letter to the address on the first page. Dr. Stacy Lindau or Dr. Elbert Huang may still use your information that was collected prior to your written notice.

You will be given a signed copy of this document. Your authorization to use and disclose your health information does not have an expiration date.

MAY WE CONTACT YOU IN THE FUTURE?
Lastly, we would like to be able to contact you in the future in the event that there are other studies that you may be interested in or eligible for. We will keep your name, telephone number, address and email address in order to re-contact you. This portion is optional; if you do not agree to be re-contacted, it does not affect your ability to be in the current study.

__ Yes, I agree to be re-contacted for future research
__ No, I do not agree to be re-contacted for future research

Initials of Subject: ___________________________
Date:

CONSENT (For participants ages 18 and older or emancipated minors):

SUBJECT
The research project and the procedures associated with it have been explained to me. The experimental procedures have been identified and no guarantee has been given about the possible results. I will receive a signed copy of this consent form for my records.

I agree to participate in this study. My participation is voluntary and I do not have to sign this form if I do not want to be part of this research study.

Signature of Subject: ______________________________
Date: ___________ Time: ___________ AM/PM (Circle)

PERSON OBTAINING CONSENT
I have explained to ______________________________ the nature and purpose of the study and the risks involved. I have answered and will answer all questions to the best of my ability. I will give a signed copy of the consent form to the subject.

Signature of Person Obtaining Consent: __________________________
Date: ___________ Time: ___________ AM/PM (Circle)
